# Factors affecting health-related quality of life following axillary lymph node dissection among breast cancer survivors in Egypt

**DOI:** 10.1038/s41598-025-21445-0

**Published:** 2025-10-10

**Authors:** Horeya Mohamed Ismail, Mostafa Ahmed Arafa, Mohamed Mostafa Tahoun, Ahmed Nabil Shama, Amr Abdel Aziz Elsaid

**Affiliations:** 1 Department of Epidemiology, High Institute of Public Health, Alexandria University, Alexandria, Egypt; 2 Department of Cancer Management and Research Development, Kafr El- Sheikh University, Kafr El- Sheikh, Egypt; 3 Department of Clinical Oncology, Faculty of Medicine, Alexandria University, Alexandria, Egypt

**Keywords:** Breast cancer survivors, Health-related quality of life, Axillary lymph node dissection, MANOVA, Cancer, Medical research, Oncology

## Abstract

Axillary lymph node dissection (ALND) remains a standard component in the management of breast cancer; however, it may have lasting impacts on patients’ health-related quality of life (HRQOL). This study aimed to assess HRQOL and identify its demographic and clinical correlates among breast cancer survivors who underwent ALND. This cross-sectional study was conducted among breast cancer survivors post-ALND at a tertiary oncology center in Alexandria, Egypt. HRQOL was measured using the Arabic FACT-B (version 4.0), and the associated factors were analyzed using multivariate analysis of variance (MANOVA). A total of 150 breast cancer survivors completed the FACT-B questionnaire, with a mean HRQOL score of 88.92 ± 18.01. Of the participants, 18.7% had poor, 64.0% had moderate, and 17.3% had good HRQOL. MANOVA showed that the type of surgery, BMI, HER2 status, lymph node involvement, and tumor stage were significantly associated with HRQOL (*p* < 0.05). Between-subject analyses and post hoc comparisons confirmed significant group differences across the FACT-B subscales. Overweight or obese participants had poorer Physical, Emotional, and Functional Well-Being. Mastectomy, HER2-positive status, and Stage III disease were associated with lower HRQOL across multiple domains, while lymph node involvement impacted the Physical Well-Being subscale only. Integrating routine HRQOL assessment into survivorship care is essential for identifying individuals at a higher risk of poor outcomes. Tailored follow-up strategies and supportive interventions for these subgroups can enhance the long-term well-being and improve the overall post-treatment quality of life of breast cancer survivors.

**Supplementary Information:**

The online version contains supplementary material available at 10.1038/s41598-025-21445-0.

## Introduction

Breast cancer (BC) is the most frequently diagnosed cancer and the leading cause of cancer-related mortality among women globally. It poses a significant and growing public health concern, with about 2.3 million new cases and over 670,000 deaths reported worldwide in 2022^1^. In Egypt, BC is the most common malignancy among women, accounting for 38.8% of cancers in that population, with the estimated number of new BC cases being nearly 22,038 in 2020, in all ages, and forecasted to be approximately 46,000 by 2050. It is estimated that the BC mortality rate is approximately 11%, making it the second leading cause of cancer-related mortality after liver cancer among the Egyptian population^2^.

Advancements in early detection and therapeutic strategies have significantly improved BC survival rates over the past few decades. As survivorship increases, there is a growing need to assess the impact of cancer diagnosis and treatment on long-term health outcomes^3^.

Health-related quality of life (HRQOL) has emerged as a critical component of comprehensive cancer care, and reflects the physical, psychological, and social challenges faced by survivors. Many of these challenges are directly associated with the disease itself and its management procedures, particularly surgical interventions such as mastectomy and lymph node clearance^4^. ALND, which involves the removal of a significant number of lymph nodes from the axilla, has been associated with a range of physical complications, including wound infections, lymphedema, lymphangitis, arm numbness, shoulder dysfunction, and restricted arm mobility. These adverse outcomes can profoundly impair survivors’ daily activities, body image, emotional well-being, and overall quality of life^5–8^.

Understanding the factors associated with impaired HRQOL is crucial for identifying high-risk patients and guiding targeted supportive interventions. Evidence shows that both demographic and clinical characteristics significantly influence HRQOL in BC survivors. Sociodemographic variables, such as age, race, and education level, contribute to disparities, highlighting the need to examine these factors across diverse populations^8,9^. Clinical and pathological factors, including tumor stage, hormone receptor status, molecular subtype, extent of surgical intervention, and adjuvant therapy, have been shown to affect long-term patient well-being^9^.

In recent years, HRQOL has become a key outcome in cancer care, guiding treatment decisions, and supporting patient-centered approaches. Among BC survivors, particularly those who have undergone ALND, HRQOL is often compromised by long-term treatment-related effects. Moreover, a growing body of evidence supports the role of HRQOL as a prognostic indicator of survival in patients with BC^10^.

While global evidence highlights the prognostic value of HRQOL in cancer populations, data from low- and middle-income countries remain limited. In Egypt, where breast cancer is the most common cancer among women, survivorship experiences may be influenced by cultural and social contexts, in addition to disease patterns and treatment practices^11^. Nevertheless, there is a notable lack of structured research addressing post-treatment quality of life and survivorship in Egypt.

This study addresses this critical gap by evaluating HRQOL among BC survivors in Egypt who previously underwent ALND. This study aimed to assess HRQOL using the validated Arabic version of the FACT-B instrument and identify its potential correlates in this population.

## Methods

### Study design and setting

This cross-sectional study was conducted at a private, specialized tertiary oncology center in Alexandria, Egypt. All BC survivors attending the oncology clinic for follow-up visits between December 2023 and December 2024 were invited to participate in the study. One of the study authors, who is the treating oncologist, identified eligible patients during their routine follow-up visits and referred them to the research team. Trained research staff then explained the study objectives, obtained written informed consent, distributed the questionnaires, and collected the completed forms before the patients left the clinic.

### Participants

Eligible patients were approached during the scheduled follow-up visits. Inclusion criteria were female patients aged 18 years or older, with a confirmed diagnosis of breast cancer, and currently in the survivorship phase (i.e., not undergoing active treatment). Patients with metastatic or recurrent disease were excluded. Recruitment was continued until the required sample size (*n* = 150) was achieved. The sample size was calculated using PASS 20.0, based on a mean score of 97.23 for HRQOL among breast cancer survivors, with a standard deviation of 20.01, considering that the difference between the null and alternative hypotheses is 4.78^12^. The minimum required sample size at 95% confidence interval and statistical power of 80% was calculated to be 138 breast cancer survivors, and it was rounded to 150 patients.

### Quality of life assessment

Health-related quality of life (HRQOL) was assessed using the validated and reliable FACT-B (version 4.0) in Arabic (https://www.facit.org). Permission to use the validated Arabic version of the FACT-B was obtained. The questionnaire was self-administered, and a trained research staff was available during data collection to provide clarification when needed, without influencing participants’ responses.

FACT-B comprises the Functional Assessment of Cancer Therapy–General (FACT-G) questionnaire, along with an additional breast cancer-specific subscale (BCS). It is a widely recognized tool for evaluating quality of life and overall well-being among breast cancer patients and has been validated across English-speaking and Arabic-speaking populations^13,14^.

The FACT-B consists of 37 items that measure five domains of HRQOL: physical well-being (PWB), social/family well-being (SWB), emotional well-being (EWB), functional well-being (FWB), and breast cancer-specific concerns (BCS).

Each domain assessed specific aspects.

- Physical well-being (PWB): symptoms such as fatigue, nausea, pain, and treatment-related side effects.
- Social/family well-being (SWB): family acceptance and support from friends.
- Emotional well-being (EWB): psychological responses and emotional concerns about illness.
- Functional well-being (FWB): the ability to perform personal and professional activities and sleep quality.
- Breast Cancer Subscale (BCS): Physical, psychological, and aesthetic concerns related to breast cancer and its treatment.

The questionnaire uses a 5-point Likert scale ranging from 0 (“not at all”) to 4 (“very much”) to assess patients’ experiences over the preceding seven days. The scores of each subscale were calculated according to the scoring manual. Scoring ranges for each domain were as follows: 0–28 for physical, social, and functional well-being subscales; 0–24 for the emotional well-being subscale; and 0–40 for the BCS. For optional items within the FACT-B questionnaire, if a respondent omitted one item, the subscale score was prorated based on the average of the answered items within that subscale. This approach was applied only when more than 50% of the items in the respective subscale were completed (e.g., at least 4 out of 7 items, or 4 out of 6 items), in accordance with the scoring guidelines of the instrument^15^.

The FACT-G comprises four domains (PWB, SWB, EWB, and FWB) with an overall score ranging from 0 to 108, where higher scores indicate better well-being. The addition of the BCS items increased the maximum overall FACT-B score to 148, with higher values indicating a more favorable quality of life. Additionally, the Trial Outcome Index (TOI) was calculated by summing the PWB, FWB, and BCS scores. The TOI provides a focused measure of physical and functional well-being and is often used as a responsive indicator of clinical changes, complementing the multidimensional FACT-B total score^16^.

### Variables

Sociodemographic variables included age, marital status, educational level, and occupation. Clinical data were extracted from the patients’ medical records and included medical history (e.g., comorbidities such as diabetes mellitus, hypertension, cardiac conditions, or other chronic diseases), prior surgical history, and family history of breast cancer. Tumor- and disease-related variables included TNM stage (I/II–III), classified according to the American Joint Committee on Cancer (AJCC) 8th edition guidelines^17^; tumor laterality (left/right breast); type of surgery (mastectomy or breast-conserving surgery (e.g., wide local excision or lumpectomy); histological type (invasive ductal carcinoma [IDC]; invasive lobular carcinoma [ILC]; or other types); tumor grade; estrogen receptor (ER) status (positive/negative); progesterone receptor (PR) status (positive/negative); Human Epidermal Growth Factor Receptor 2 (HER2) status (positive/negative); Lymph Node (LN) status (positive/negative); number of disected LN (≤10, > 10), and and details of adjuvant therapies.

### Statistical analysis

All statistical analyses were performed using SPSS version 26 (IBM Corp., Armonk, NY, USA). Descriptive statistics were reported as frequencies, means, and standard deviations. HRQOL scores, including subscales and total scores (TOI, FACT-G, and FACT-B), were summarized using means, standard deviations, and 95% confidence intervals (CI) around the mean. To facilitate interpretation, we converted the mean scores to percentages that indicate the average percentage of the score relative to the total score (i.e., dividing the observed mean score by the total possible score and multiplying by 100). Based on thresholds used in previous studies, the overall HRQOL was classified as poor (< 50%), moderate (50–70%), or good (> 70%)^18^.

The distribution of subscale scores and the total FACT-B score were assessed for normality using normality plots and statistical tests. Multivariate Analysis of Variance (MANOVA) was conducted to test the hypothesis of a significant association between a set of interrelated dependent variables (the HRQOL subscales) and various independent variables. The significance level (α) for the MANOVA was set at 0.05. Post-hoc analysis using Tukey’s test was performed to identify which HRQOL subscales showed significant group differences and to determine the specific groups between which these differences existed.

## Results

### Sample characteristics

A total of 150 women completed the FACT-B. Their sociodemographic characteristics are summarized in Table A (Appendix 1). The mean age of participants was 57.61 ± 11.31 years (range: 31–87 years). The average time since diagnosis at the time of questionnaire administration was 48 months (range, 24– 84 months). Most of the participants were married (78.7%) and residing in urban areas (91.3%). Most participants had completed university or postgraduate education (62.7%). Regarding employment status, 55.3% were housewives, 26.0% were employed, and 18.7% were retired. Additionally, 62.7% of the participants were postmenopausal, 78.7% had multigravida, 46.7% had at least one comorbidity, 54.7% reported a history of surgery, and 25.3% reported a family history of breast cancer.

The clinical and pathological features of the tumor are presented in Table 1. Over half of the patients (52.7%) underwent mastectomy, while 47.3% underwent conservative breast surgery (e.g., lumpectomy). Many tumors were hormone receptor–positive, with 86.0% being ER–positive and 77.3% PR–positive, and 18.7% had overexpressed HER2. More than half (59.3%) of the patients had lymph node involvement. Regarding molecular subtypes, Luminal A (HR^+^/HER2^–)^ was the most prevalent (72.0%), followed by Luminal B (HR^+^/HER2^+^) at 14.7%, triple-negative (HR^−^/HER2^−^) at 9.3%, and HER2-enriched (HR^–^/HER2^+^) at 4.0%. Based on TNM pathological staging, 7.3% of the patients were at stage I, 64.7% at stage II, and 28.0% at stage III.

**Table 1:** Distribution of the clinico-pathological factors of the study participants.

Characteristics	Frequency (%)
Time since diagnosis (months)	
Median (IQR)	48 (24–84)
Minimum - maximum	(12–180)
Type of Surgery	
MRM	79 (52.7)
BCS	71 (47.3)
Laterality	
Right	84 (56.0)
Left	66 (44.0)
Histological type	
IDC	130 (86.7)
ILC	20 (13.3)
Histological grade	
I	3 (2.0)
II	128 (85.3)
III	19 (12.7)
ER	
Positive	129 (86.0)
Negative	21 (14.0)
PR	
Positive	116 (77.3)
Negative	34 (22.7)
HER2	
Positive	28 (18.7)
Negative	122 (81.3)
Lymph Node status	
Positive	89 (59.3)
Negative	61 (40.7)
Total number of disected LN	
≤10	65 (43.3)
> 10	85 (56.7)
Subtype	
HR^+^/HER2^−^	108 (72.0)
HR^+^/HER^+^	22 (14.7)
HR^−^/HER^+^	6 (4.0)
HR^−^/HER^−^	14 (9.3)
AJCC Stage	
Stage I	11 (7.3)
Stage II	97 (64.7)
Stage III	42 (28.0)
Type of Adjuvant Therapy	
Chemotherapy	86 (59.3)
Radiotherapy	89 (59.3)
Hormonal Therapy	119 (79.3)
Targeted Therapy	24 (16.0)

### Health-related quality of life

Table 2 presents the mean scores, standard deviations, and 95% confidence intervals for the five FACT-B subscales and the TOI, FACT-G, and FACT-B total scores. Corresponding percentage scores were included to facilitate interpretation. Among the subscales, Physical Well-Being showed the highest mean score (66.42%), followed closely by Emotional Well-Being (65.66%), while Functional Well-Being (55.32%) and the Breast Cancer Subscale (55.65%) had the lowest scores, indicating greater impairment in these areas. The Social/Family Well-Being score was also relatively low at 59.96%. The overall TOI, FACT-G, and FACT-B mean scores were 58.69%, 61.71%, and 60.08%, respectively, reflecting a moderate level of HRQOL among participants. In terms of overall HRQOL levels, 18.7% of participants reported poor, 64% reported moderate, and 17.3% reported good scores of HRQOL.

**Table 2:** Descriptive statistics of FACT-B subscale and total scores.

Scale	Descriptives Mean ± SD (95% CI)	Mean scores Percentage
PWB Subscale score Scoring range (0–28)	18.60 ± 4.72 (17.84–19.36)	66.42%
SWB Subscale score Scoring range (0–28)	16.79 ± 5.78 (15.86–17.73)	59.96%
EWB Subscale score Scoring range (0–24)	15.76 ± 3.94 (15.13–16.40)	65.66%
FWB Subscale score Scoring range (0–28)	15.49 ± 4.86 (14.71–16.27)	55.32%
Breast Cancer Subscale (BCS) Scoring range (0–40)	22.26 ± 5.04 (21.44–23.07)	55.65%
FACT-B Trial Outcome Index (TOI) (0–96)	56.35 ± 11.68 (54.46–58.23)	58.69%
FACT-G Total score Scoring range (0–108)	66.65 ± 14.69 (64.28–69.03)	61.71%
FACT-B total score Scoring range (0–148)	88.92 ±18.01 (86.01–91.82)	60.08%
FACT-B Score Level, n (%)		
Poor < 50%	28 (18.7)	
Moderate 50–70%	96 (64.0)	
Good > 70%	26 (17.3)	

### Factors associated with health-related quality of life

Multivariate analysis of variance (MANOVA) was conducted to examine the influence of various demographic and disease-related variables on the five subscales of the FACT-B, representing different dimensions of health-related quality of life (HRQOL) among breast cancer survivors. Normality testing using Shapiro–Wilk tests (*p* > 0.05) and inspection of normality plots indicated that the total FACT-B score and all subscale scores were approximately normally distributed. The dependent variables included Physical Well-Being, Social/Family Well-Being, Emotional Well-Being, Functional Well-Being, and the Breast Cancer Subscale.

The overall MANOVA revealed a statistically significant multivariate effect of five variables: BMI (F = 1.993, *p* = 0.034), type of surgery (F = 2.191, *p* < 0.05), HER2 status (F = 2.930, *p* = 0.015), lymph node status (F = 2.333, *p* = 0.045), and tumor stage (F = 2.430, *p* = 0.009) were all significantly associated with HRQOL. The corresponding partial eta-squared values, indicating the proportion of total variability attributable to each factor, ranged from 0.065 to 0.101. The highest effect size was observed for disease stage (0.78), followed by type of surgery (0.101), while the lowest was for BMI (0.065). Table 3 .

**Table 3:** Multivariate analysis of variance (MANOVA) identifying the factors affecting HRQOL of breast cancer survivors following ALND (n = 150).

Factor	F Statistic	*P*- value	Partial eta squared
Age	0.852	0.619	0.029
Marital Status	0.223	0.952	0.008
Residence	0.884	0.493	0.03
Education	1.031	0.417	0.035
Occupation	0.487	0.898	0.017
Menopausal Status	0.797	0.553	0.027
BMI	1.993	0.034*	0.065
Gravidity	0.282	0.922	0.01
Comorbidities	2.053	0.075	0.067
Surgical History	1.367	0.240	0.045
Family history of BC	1.850	0.107	0.06
Type of Surgery	3.228	0.009*	0.101
Laterality	1.337	0.252	0.044
Histological type	1.034	0.420	0.035
Histological grade	1.004	0.450	0.034
ER	1.178	0.323	0.039
PR	1.310	0.263	0.044
HER2	2.930	0.015*	0.092
LN status	2.333	0.045*	0.075
Number of discted LN	1.709	0.136	0.056
Stage	2.430	0.009*	0.78
Subtype	1.421	0.134	0.048

Statistical significance at (*P* < 0.05).

Following the significant multivariate effects identified in the MANOVA, between-subjects analyses were conducted to determine which specific FACT-B subscales were influenced by the independent variables. The results in Table 4 showed that BMI had a significant impact on the Physical, Emotional, Functional Well-Being, and Breast Cancer subscales. Both the type of surgery and HER2 status were also influential; patients who underwent mastectomy and those with positive HER2 status reported significantly lower scores across all subscales compared to those who had breast-conserving surgery or negative HER2 status. Positive lymph node involvement was associated with lower scores in the Physical Well-Being domain only. In addition, advanced tumor stage was significantly associated with lower mean scores on the Physical Well-Being, Emotional Well-Being, Breast Cancer Subscale, and total FACT-B score. Obese women reported a significantly lower score for all five dimensions and the total score. For the BC stage, stage III survivors reported a significantly lower score for all scales except for social and functional well-being.

**Table 4:** Between-Subjects Effects of Clinical and Demographic Factors on HRQOL Subscales and Total FACT-B Score.

Factor	PWB	SWB	EWB	FWB	BCS	FACT-B Total
BMI, P-value	**0.005**	0.177	**0.015**	**0.004**	**0.002**	**0.001**
Healthy mean±SD	19.78±4.43	18.31±7.42	16.60±5.06	17.26±5.13	23.52±6.57	95.48±23.61
Overweight mean±SD	20.04±3.93	17.57±5.37	17.11±3.77	17.05±4.48	24.43±4.27	96.57±15.76
Obese mean±SD	17.61±4.83	16.12±5.41	15.04±3.53	14.45±4.68	21.12±	84.36±15.77
Type of Surgery P-value	**0.001**	**0.012**	**0.001**	**0.006**	**0.014**	*P* < 0.001
Mastectomy mean±SD	17.42±4.71	15.67±5.43	14.81±3.58	14.47±4.89	21.30±4.73	83.67±16.50
Lumpectomy mean±SD	19.92±4.41	18.04±5.93	16.83±4.06	16.64±4.59	23.32±5.21	94.74±17.91
HER2 status P-value	**0.037**	**0.004**	**0.004**	**0.001**	**0.033**	*P* < 0.001
Negative mean±SD	18.98±4.55	17.45±5.82	16.20±3.71	16.12±4.89	22.68±5.03	91.42±17.46
Positive mean±SD	16.92±5.14	13.97±4.70	13.85±4.38	12.82±3.73	20.42±4.80	78.01±16.43
LN status P-value	**0.01**	0.304	0.607	0.997	0.691	0.576
Negative mean±SD	19.78±4.40	16.21±6.39	15.96±4.19	15.49±5.08	22.46±5.32	89.92±19.53
Positive mean±SD	17.79±4.78	17.20±5.32	15.63±3.77	15.49±5.08	22.123±4.86	88.23±16.95
Stage P-value	*P* < 0.001	0.834	**0.028**	0.273	**0.003**	**0.014**
Stage I mean±SD	20.0±4.56	16.75±3.70	16.36±4.43	15.64±4.52	23.09±4.81	91.85±14.16
Stage II mean±SD	19.59±4.24	16.60±6.22	16.30±3.92	15.93±5.27	23.16±4.88	91.58±1.912
Stage III mean±SD	16.02±4.88	17.74±5.24	14.42±3.59	14.49±3.83	20.05±4.91	82.22±14.51

Statistical significance at (*P* < 0.05).

**Fig. 1:**
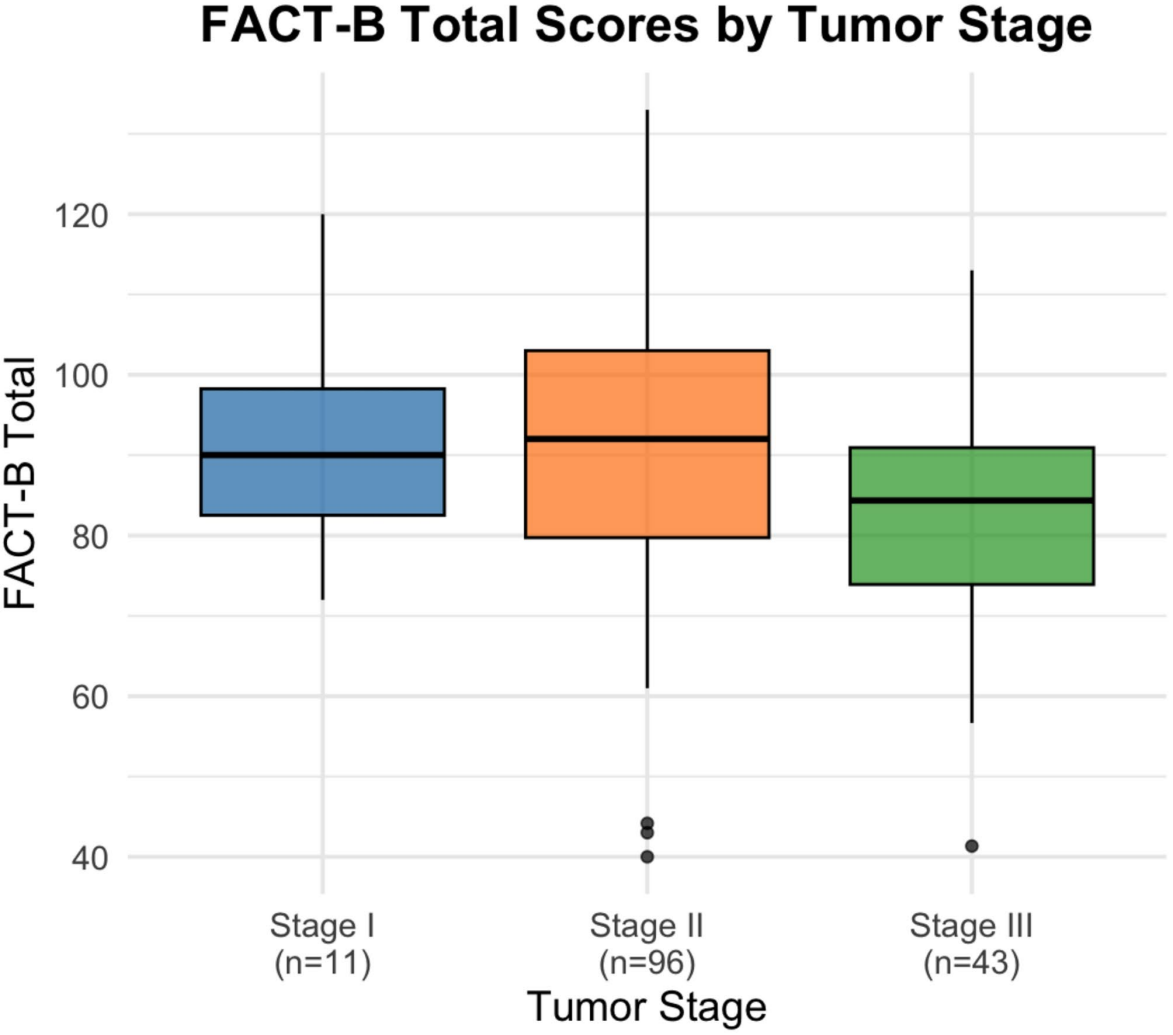
Overall Quality of Life, Measured by FACT-B, by Tumor Stage Among Breast Cancer Survivors in Egypt. Boxplots of FACT-B total scores by tumor stage (Stage I–III). Boxes indicate the interquartile range (IQR), horizontal lines represent medians, whiskers extend to 1.5 × IQR, and points beyond whiskers are outliers. Post-hoc analysis was performed using Tukey’s test.

Figure 1 illustrates FACT-B total scores across tumor stages. Patients with Stage III tumors exhibited a clear decline in overall quality of life compared to those with Stage I and II disease, while scores for the earlier stages were relatively similar.

For independent variables with more than two levels, post hoc analyses were conducted to identify specific group differences in the FACT-B subscale scores. Table 5 For BMI, overweight or obese participants reported significantly lower scores on the PWB, EWB, and BCS than those with a normal BMI. However, no statistically significant differences were observed between the overweight and obese groups in these subscales. In contrast, the FWB subscale and total FACT-B score demonstrated significant differences across all BMI categories. Specifically, obese participants scored significantly lower than both overweight and normal-weight participants, and overweight participants had significantly lower scores than those in the normal-weight group.

**Table 5:** Post-Hoc Comparisons of HRQOL Subscale Scores by BMI Category and Tumor Stage.

Independent Variable	Subscale	Group comparison	*P*- Value^*^
BMI	PWB	Obese vs. Normal & Overweight vs. Normal	0.008
	EWP	Obese vs. Normal & Overweight vs. Normal	0.021
	FWB	Obese vs. Overweight Obese vs. Normal & Overweight vs. Normal	0.031, 0.017
	BCS	Obese vs. Normal & Overweight vs. Normal	0.002
Stage	PWB	Stage I vs. Stage III, Obese vs. Normal & Overweight vs. Normal	0.025, *P* < 0.001
	EWB	Stage II Vs Stage III	0.024
	BCS	Stage II Vs Stage III	0.002

*Only significant groups (*P* < 0.05) are displayed.

For BC stage, results revealed that participants who had Stage III reported significantly lower PWB scores than those who had Stage I (*p* = 0.025) and Stage II (*p* < 0.001), indicating a decline in physical health with more advanced disease. However, for EWB, BCS, and total FACT-B scores were significantly lower in Stage III patients than in Stage II patients only, indicating a marked decline in quality of life at more advanced disease stages.

## Discussion

Recent advances in early detection and therapeutic strategies have significantly improved survival rates among women with BC, gradually shifting the disease from a life-threatening to a more chronic and manageable condition. This progress has resulted in a growing population of survivors and a new clinical imperative: to move beyond simply extending disease survival toward enhancing the quality of life during survivorship^3^. In this context, our study aimed to assess HRQOL outcomes and associated factors among breast cancer survivors in Egypt, with an average time since diagnosis of four years. Our findings reflect the enduring impact of both the disease and its management, particularly invasive procedures such as ALND, across multiple dimensions of quality of life.

Our results indicated that 64% of the sample had moderate HRQOL, while 18.7% experienced poor HRQOL and only 17.3% achieved good HRQOL. The mean FACT-B total score in our cohort was 88.92 ± 18.01, which is lower than the scores reported in Western populations^19–21^. This discrepancy may be attributed to differences in healthcare access, socioeconomic conditions, and the availability of structured survivorship care.

Survivorship care infrastructure differs substantially between high-income countries (HICs) and low- and middle-income countries (LMICs) like Egypt. In HICs, structured survivorship programs and wider access to breast-conserving surgery, reconstruction, and psychosocial support have been shown to improve HRQOL. In contrast, care in Egypt is often limited to recurrence surveillance, with minimal rehabilitation or psychosocial interventions. Gaps are particularly evident in lymphedema care, where standardized screening and early interventions are routine in HICs but remain scarce in Egypt^22^. Additionally, gender dynamics significantly influence survivorship experiences, as Egyptian women often prioritize family responsibilities over their own health needs, leading to underreporting of symptoms and reduced engagement with follow-up care^23,24^. Financial barriers and limited insurance coverage also impose substantial economic burden compared with HICs^25^. Collectively, these contextual factors may explain the persistent moderate-to-poor HRQOL observed in our cohort and highlight why survivorship interventions developed in HICs cannot be directly transplanted to LMICs without adaptation. Resource-sensitive models, including community health workers and telehealth, show promise in bridging this gap^26^.

While previous research has shown that HRQOL may decline during active treatment^21,27^, our results emphasize that these challenges can persist well into survivorship and many years after diagnosis, especially in resource-limited settings. These findings indicate that the consequences of BC persist well beyond the completion of active treatment and highlight the urgent need for comprehensive survivorship care plans.

While MANOVA identified factors significantly associated with HRQOL, the observed differences in FACT-B scores also appear clinically meaningful. Prior studies suggest that a 7–8 point change in total FACT-B score represents a minimally important difference^28^. In our cohort, differences across BMI, surgery type, HER2 status, and disease stage exceeded this threshold. For instance, obese survivors scored 11 points lower than those with healthy BMI, and mastectomy patients scored 11 points lower than lumpectomy patients. These findings highlight actionable areas in survivorship care, including weight management, surgical counseling, targeted support for HER2-positive patients, and enhanced psychosocial care for advanced-stage survivors. Taken together, our results reveal that HRQOL disparities are not only statistically detectable but also clinically meaningful, reinforcing the need for individualized survivorship strategies in resource-limited settings.

Our findings revealed significant variations in the levels of impairment across the different domains of HRQOL, highlighting the diverse challenges faced by breast cancer survivors. Physical Well-Being (66.42%) and Emotional Well-Being (65.66%) had the highest mean percentage scores, suggesting relatively better preservation of physical function and emotional coping among participants. These results are consistent with previous studies suggesting that many BC survivors experience gradual improvements in physical recovery and emotional coping following the completion of active treatment^29,30^. However, further analysis identified specific subgroups more vulnerable to physical impairment, including overweight and obese participants, as well as those with adverse clinicopathological features such as mastectomy, HER2-positive status, lymph node involvement, and advanced-stage disease. These findings align with those a of systematic review by Klein et al. (2023), which identified ALND, high BMI, mastectomy, advanced disease stage, and adjuvant therapies as major risk factors for long-term arm morbidity. Klein et al. emphasized that recognizing these factors is essential for tailoring early rehabilitation and follow-up care to prevent or mitigate long-term physical morbidity^31^. Our results similarly emphasize the importance of proactive, targeted support to maintain physical and emotional well-being in high-risk survivors.

In addition, our study assessed the magnitude of the observed effects using partial eta squared values. The highest effect size was observed for disease stage (0.78), followed by type of surgery ( 0.101), while the lowest was for BMI (0.065). This indicates that disease stage has a very large impact on HRQOL, type of surgery has a moderate effect, and BMI and other factors have smaller effects. Clinically, this suggests that interventions aimed at patients with advanced-stage disease are likely to yield the greatest improvements in quality of life. Surgical decision-making and post-operative support should also be carefully considered, while weight management and lifestyle interventions may provide additional benefits.

In our study, functional well-being and BCS recorded the lowest scores, reflecting substantial and persistent impairments in daily functioning and BC-specific concerns. Functional Well-Being, which encompasses the ability to perform personal and occupational activities, sleep quality, and overall life role participation, appears particularly vulnerable during long-term survivorship.

Other studies have reported similar challenges, where survivors often struggle to fully return to their pre-diagnostic functional capacity, particularly in relation to fatigue, pain, and sleep disruption^32^. A 15-year longitudinal study further supports this finding, revealing that although overall HRQOL tends to improve over time, survivors may continue to experience long-term functional limitations^33^.

Interventions aimed at improving functional capacity have shown promise in addressing these challenges. Physical activity, including aerobic and resistance exercises, has been demonstrated to significantly reduce cancer-related fatigue, improve sleep quality, and enhance the overall quality of life among breast cancer survivors^34,35^.

The social/family well-being score was also relatively low in our study, suggesting that BC survivors in Egypt may encounter significant difficulties in maintaining social relationships and family support during survivorship. Our analysis revealed that this subscale was most negatively affected among patients who underwent mastectomy and those with HER2-positive disease, which is often associated with more aggressive disease and intensive treatment regimens. Previous research has shown that extensive surgical procedures and biologically aggressive tumors can exacerbate body image concerns, reduce self-esteem, and contribute to perceived social withdrawal and disruption of sexual relationships. These factors may compound feelings of isolation and hinder reintegration into social roles^36,37^.

Social support has been widely reported to play a key role in improving the psychosocial burden of diseases. Leung et al. found that higher levels of perceived social support were significantly associated with better mental and physical health-related quality of life among patients with BC, while lack of support was linked to increased emotional distress and delayed recovery^38^. Similarly, Belau et al. studied the long-term effect of social realtionships on HRQOL and found that breast cancer survivors^31^ with strong social networks maintained better overall health status even 15 years post-diagnosis. These studies emphasized the lasting influence of social connections on survivorship outcomes. These findings highlight the importance of incorporating social support interventions into survivorship care, particularly for patients with more invasive surgeries or aggressive tumor subtypes.

Finally, the low score on the Breast Cancer Subscale reflects issues unique to breast cancer survivorship, including altered body image, concerns about femininity, fear of recurrence, arm swelling, and decreased sexual attractiveness. These concerns are well-documented in the literature to be relevant to BC rather than other cancers because of the direct impact of treatment that is often exacerbated by procedures like mastectomy and ALND on physical appearance and sexual identity^40,41^.

In conclusion, our results emphasize the importance of integrating HRQOL assessment into routine follow-up for BC survivors, particularly those undergoing ALND. Targeted interventions, such as physical rehabilitation for lymphedema, psychosocial support for patients undergoing mastectomy, and lifestyle programs addressing weight management, may help mitigate the negative effects identified in this study. These findings highlight the need for multidisciplinary survivorship. Additionally, we emphasize the need for culturally tailored, patient-centered survivorship care strategies that address the specific challenges faced by this population. Future research should build on these findings by developing and evaluating targeted interventions aimed at improving long-term quality of life outcomes, particularly for patients with a higher BMI, advanced-stage disease, or those undergoing more invasive treatments.

### Strengths and limitations

A key strength of this study was its focus on an underrepresented population. To our knowledge, this is the first study to evaluate HRQOL among breast cancer survivors post-ALND in Egypt, and one of the few conducted in Arab populations. This adds valuable evidence to the limited body of survivorship research in the Middle East, emphasizing the importance of assessing HRQOL within specific cultural, social, and healthcare system contexts.

However, this study has some limitations. This study was conducted at a single tertiary care center, which may introduce selection bias, as women attending follow-up at a private center may not fully represent the broader Egyptian or regional breast cancer survivor population, thereby limiting generalizability. Given the cross-sectional design, we were also unable to assess baseline HRQOL or track changes over time, limiting causal interpretations and the ability to determine the direct impact of ALND. Future research using a longitudinal approach with repeated assessments would allow for a clearer understanding of how HRQOL evolves throughout survivorship and the long-term impact of interventions. Lastly, the absence of country-specific normative data for the FACT-B instrument limits its ability to calculate standardized composite scores and compare results with international populations. The development of locally validated norms would greatly enhance the interpretability and relevance of future research.

## Supplementary Information

Below is the link to the electronic supplementary material.

Supplementary Material 1

## Data Availability

The datasets used and/or analysed during the current study available from the corresponding author on reasonable request.
