# Supplementary material for: Factors affecting health-related quality of life following axillary lymph node dissection among breast cancer survivors in Egypt
Source: Sci Rep. 2025 Oct 10;15:35402. doi: 10.1038/s41598-025-21445-0 (PMC12513989; doi:10.1038/s41598-025-21445-0)
Supplement: Supplementary file 1 — Supplementary Material 1 [file 41598_2025_21445_MOESM1_ESM.docx]

**Results**

**Table (A): Socio-demographic characteristics and medical history of the study participants**

| **Characteristics** | **Frequency (%)** |
| --- | --- |
| **Age**  **Minimum- Maximum**  **Mean (SD)**  30 – 40  41 – 50  51 – 60  >60 | **31 – 87**  **57.61(11.31)**  9 (6.0)  39 (26.0)  34 (22.7)  68 (45.3) |
| **Marital Status**  Married  Not married | 118(78.7)  32 (21.3) |
| **Residence**  Urban  Rural | 137(91.3)  13(8.7) |
| **Education**  Elimintary  Secondary  University/ Postgraduate | 9 (6.0)  47 (31.3)  94 (62.7) |
| **Occupation**  Housewife  Employed  Retired | 83 (55.3)  39 (26.0)  28 (18.7) |
| **Menopausal Status**  Pre-menopause  Menopause | 56 (37.3)  94 (62.7) |
| **Gravidity**  Nullgravida  Multigravida | 32 (21.3)  118(78.7) |
| **Comorbidities**  Yes  No | 70 (46.7)  80 (53.3) |
| **Surgical History**  Yes  No | 82 (54.7)  68 (45.3) |
| **Family history of BC**  Yes  No | 38 (25.3)  112 (74.7) |
